# Nontargeted metabolomics uncovering metabolite signatures in glioblastoma: a preliminary study on candidate biomarker discovery for IDH subtyping and survival prediction

**DOI:** 10.3389/fonc.2025.1568040

**Published:** 2025-05-08

**Authors:** Peng Xu, Xiling Chen, Qun Li, Zheqing Dong, Ji Zhu, Zhipeng Su, Qifan Zhang, Kui Fang

**Affiliations:** ^1^ Clinical Laboratory, The Third Affiliated Hospital of Zhejiang Chinese Medical University, Hangzhou, China; ^2^ Department of Neurosurgery, First Affiliated Hospital of Wenzhou Medical University, Wenzhou, China

**Keywords:** glioblastoma, isocitrate dehydrogenase, molecular classification, biomarkers, survival risk prediction

## Abstract

**Background:**

Currently, there are no established tumor-derived metabolic biomarkers in clinical practice that can simultaneously differentiate among nontumorous brain tissues, isocitrate dehydrogenase (IDH) wild-type glioblastomas (GBMs), and IDH mutant GBMs, or accurately predict patient survival. The aim of this study was to identify GBM biomarkers for molecular classification and survival prediction via nontargeted metabolomics.

**Methods:**

Brain tissue samples from nontumors, IDH-mutant GBMs, and IDH-wild-type GBMs were analyzed via liquid chromatography-mass spectrometry (LC–MS). Metabolites for molecular classification and survival prediction were identified via sparse partial least-squares discriminant analysis (sPLS–DA) and extreme gradient boosting (XGBoost) models, respectively. Both sets of metabolites were then validated via bootstrap resampling. The biomarkers for survival prediction were further validated using an independent metabolomics dataset.

**Results:**

In total, 185 human-derived metabolites were identified with high confidence levels. Two non-overlapping sets of 11 candidate biomarkers for molecular subtyping and survival prediction were screened out. In the validation models for molecular subtyping, the random forest model achieved the highest accuracy (0.787, 95% CI: 0.780–0.795) and a Kappa value of 0.681. The Cox proportional hazards regression model established based on cholic acid and citrulline had an AUC of 0.942 (95% CI: 0.920-0.956) at 84 days and an AUC of 0.812 (95% CI: 0.746-0.826) at 297 days.

**Conclusion:**

This exploratory study identified potential metabolic biomarkers for GBM subtyping and prognosis prediction. However, further validation in large-scale clinical studies and mechanistic investigations are needed to confirm their applicability and reliability.

## Introduction

Glioblastomas (GBMs) constitute the most aggressive and swiftly progressing form of malignant brain tumor, accounting for the majority of deaths associated with brain cancer [1]. Progress in comprehending the genetic and epigenetic modifications of GBM has revealed substantial heterogeneity, characterized by distinct molecular subtypes, such as the isocitrate dehydrogenase (IDH)-mutant (approximately 10%, originating from low-grade glioma) and IDH-wildtype (approximately 90%) subtypes (1). Different molecular subtypes of GBM exhibit distinct potential therapeutic targets and prognoses, but even patients with the same subtype demonstrate significant variations in prognosis (2–7), indicating the need for personalized treatment regimens for GBM. Nonetheless, the recommended treatment regimen for all GBMs entails gross total resection (GTR) accompanied by radiotherapy and chemotherapy, irrespective of the molecular subtype and prognosis. Two critical factors that impede the implementation of personalized treatment are the difficulty in precisely determining the molecular subtype of GBM preoperatively and the challenge in forecasting the patient’s postoperative survival risk.

Metabolites can provide highly specific and sensitive indicators of cellular processes and physiological states within GBM, making them promising diagnostic and prognostic biomarkers. Numerous studies have demonstrated significant metabolic heterogeneity among tumor cells and immune cells within GBM tumor masses across different molecular subtypes (8, 9). Even within the same molecular subtype of GBM, heterogeneity has been observed (10). These metabolic changes are closely related not only to the invasiveness, survival ability, and drug resistance of glioblastoma (GBM) cells but also to patient survival (8, 11, 12). Therefore, it is feasible to identify reliable molecular and prognostic biomarkers from metabolites. Previously, Ferrasi et al. identified a set of markers in serum associated with early-stage GBM (13), whereas Shen et al. identified a set of markers related to patient survival risk (14). These studies are highly important for the early diagnosis and prognosis prediction of GBM. However, they also noted that these metabolic changes might not be attributable to tumor cells, potentially compromising the tumor specificity of these diagnostic markers. Studies on 2-hydroxyglutarate (2-HG), a classic marker of IDH-mutated GBM, have shown that this intracellular biomarker also has excellent discriminative ability in cerebrospinal fluid (15). The above research results demonstrate that markers derived from tumor tissues have the potential to serve as biomarkers in bodily fluids, and moreover, markers in tissues exhibit greater tumor specificity.

The primary aim of this study was to screen preoperative diagnostic biomarkers derived from tumors to distinguish between nontumor brain tissues, IDH-mutant GBM, and IDH-wild-type GBM through metabolomic analysis. Additionally, a cohort of biomarkers has been identified to predict the survival risk of patients after GTR. To our knowledge, this is the first study to investigate biomarkers for three-category classification (nontumor, IDH-mutant, and IDH-wild-type GBM) and survival risk prediction via nontargeted metabolomic analysis of brain tissue samples. These metabolites not only are crucial for elucidating the metabolic characteristics of glioblastoma but also hold potential as candidate biomarkers for large-scale validation in cerebrospinal fluid and other body fluids in future studies.

## Methods and materials

### Patient selection

This study was approved by the Research Ethics Committee of the First Affiliated Hospital of Wenzhou Medical University (approval number 2022-643) and conducted in accordance with the guidelines of the Declaration of Helsinki. All patients signed an informed consent form before surgery. Thirty brain tissue samples were randomly collected, including 10 from nontumor conditions, 10 from IDH-mutant GBM, and 10 from IDH-wildtype GBM, at The First Affiliated Hospital of Wenzhou Medical University between April 2016 and December 2023. The diagnosis and classification of patients with glioblastoma were based on the fourth edition of the WHO Classification of Tumors of the Central Nervous System (WHO CNS4). All patients with GBM underwent gross tumor resection (GTR). The brain tissue samples were stored in liquid nitrogen (−196°C) until metabolite extraction and analysis.

### Sample preparation

The samples were then ground in liquid nitrogen. To 20 mg of the ground sample, 400 μL of a solution (methanol:water = 7:3, v/v) containing an internal standard was added, and the mixture was vortexed at 1500 rpm for 5 min. After incubation on ice for 15 min, the samples were centrifuged at 12,000 rpm for 10 min at 4°C. A 300 μL aliquot of the supernatant was collected and stored at -20°C for 30 min. The sample was then centrifuged again at 12,000 rpm for 3 min at 4°C. Finally, a 200 μL aliquot of the supernatant was transferred for liquid chromatography-mass spectrometry (LC–MS) analysis.

### LC and MS conditions

The samples were analyzed in a blinded manner via two LC–MS methods: one under positive-ion conditions and the other under negative-ion conditions. Both methods employed a Waters ACQUITY Premier HSS T3 column (1.8 µm, 2.1 mm × 100 mm) with a gradient elution of 0.1% formic acid in water (solvent A) and 0.1% formic acid in acetonitrile (solvent B). The analytical conditions included a column temperature of 40°C, a flow rate of 0.4 mL/min, and an injection volume of 4 µL. MS analysis was conducted in information-dependent acquisition (IDA) mode via Analyst TF 1.7.1. The source parameters were set as follows: GAS1/GAS2 at 50 psi, CUR at 25 psi, TEM at 550°C, DP at ± 60 V, and ISVF at ± (5000/4000) V for positive/negative modes, respectively. The TOF–MS scan parameters included a mass range of 50–1000 Da, an accumulation time of 200 ms, and dynamic background subtraction. The product ion scan parameters included a mass range of 25–1000 Da, accumulation time of 40 ms, collision energy of ±30 V, collision energy spread of 15 V, resolution of UNIT, charge state of 1, intensity threshold of 100 cps, isotopes excluded within 4 Da, mass tolerance of 50 ppm, and a maximum of 18 candidate ions monitored per cycle. Detailed information on the instruments, reagents, and conditions used in this study are provided in S2 **Supplementary Tables S1–S4**.

### Data preprocessing

The raw mass spectrometry data were preprocessed via ProteoWizard, and peak detection, alignment, and retention time correction were performed using the XCMS program. Peaks with a missing rate exceeding 50% across all samples were filtered out, and missing values were imputed via the K-Nearest Neighbors (KNN) method. The peak areas were normalized via support vector regression (SVR). Metabolites with a combined identification score greater than 0.5 and a coefficient of variation (CV) of less than 0.3 in the quality control (QC) samples were accepted for further analysis. The mass spectrometry data were first subjected to log transformation, correlation filtering (threshold = 0.9), and zero-variance filtering to minimize unnecessary computations. Only human-derived metabolites that had been explicitly identified at level 1 were used to screen for biomarkers. The origins of the metabolites were determined via the MetOrigin 2.0 platform (18), which retrieves information from seven widely recognized databases.

### Identification of candidate biomarkers for molecular subtypes

This was accomplished by tuning and establishing a sparse partial least squares discriminant analysis (sPLS-DA) method using the package *mixOmics* version 6.26.0 (19). All analyses were conducted via R software (version 4.4.2). During the tuning process, the optimal number of components and variables (metabolites) within each component were determined through a grid search that explored all possible parameter combinations. Metabolites were selected as biomarkers based on their contribution to the model. The performance of the biomarkers was validated via six classic algorithms: Decision Tree, Random Forest, Neural Network, Conditional Inference Tree, C5.0 Decision Tree, and Support Vector Machine (using the *caret* 6.0.94 package). The validation process was completed by 1,000 bootstrap resampling iterations. The confusion matrices, receiver operating characteristic (ROC) curves, and area under the curves (AUCs) were used to evaluate the performance of the validation models. Note that when the predicted value represents a probability, a threshold of 0.5 is typically employed to differentiate between positive and negative outcomes.

### Identification of candidate biomarkers for survival risk prediction and validation

Survival analysis was conducted using the extreme gradient boosting (XGBoost) algorithm, with the Cox proportional hazards regression model serving as the objective function and negative log-likelihood as the evaluation metric (using the package *XGBoost* 1.7.8.1). The model was trained until the negative log-likelihood coefficient failed to decrease for ten consecutive training epochs. To comprehensively evaluate the impact of the biomarkers, Cox models were established separately via ordered combinations of the top biomarkers, and the C-index of each model was calculated to assess its predictive performance. Additionally, time-dependent ROC curves were used to evaluate the performance of the Cox model, which was fitted by the top two candidate biomarkers, via 200 bootstrap resampling iterations. The Top markers significantly associated with survival risk were validated using an independent metabolomics dataset of GBM (MTBLS3873 from Metabolights database). Twenty-five patients diagnosed with glioblastoma (Grade IV) were filtered out from the validation dataset, among whom 1 patient had an IDH mutation and 24 patients were of the wild-type.

### Characteristic metabolite analysis

The Games–Howell method was employed for significance testing of biomarkers across different groups, accommodating data that were not normally distributed and exhibited unequal variances. The Holm–Bonferroni method was used to adjust the P values for multiple comparisons. In the analysis of the impact of biomarkers on overall survival (OS), cutoff values for marker levels were determined via the maximally selected rank statistics method tailored to OS outcomes (using the R package *survival* 3.7). The survival function was estimated via the Kaplan–Meier method, and survival curves were plotted using the *survminer* package (version 0.4.9). Time-dependent ROC curves were estimated via the inverse probability of censoring weighting (IPCW) method, without considering competing risks, to evaluate the performance of the Cox proportional hazards regression models. The variation in the analysis results is represented via a 95% confidence interval (95% CI).

## Result

### Patients characteristics

The control (CTRL) group comprised three female and seven male nontumor patients aged between 30 and 75 years (median age: 52 years). Diagnoses in the nontumor group included cerebral hemorrhage, vascular malformations, moyamoya disease, and epilepsy. The IDH mutation (IDH) group consisted of four male and six female patients with IDH-mutant GBM, with a median age of 37.5 years (range: 30–52 years). The IDH-wild-type (WT) group included eight male and two female patients with IDH-wild-type GBM, with a median age of 59 years (range, 43–75 years). The male-to-female ratio among patients ranged from 60% to 40%. The median OS was 99 weeks (95% CI: 29.3–395.5). Other clinical information of the patients is presented in S1 **Supplementary Table S1**.

### Metabolites identification

A total of 2533 metabolites were identified from the 30 samples, of which 549 were unequivocally identified at level 1. Among the 549 metabolites, 185 were of human origin. The detailed quality control results for LC–MS are provided in S2 **Supplementary Figures S1–S3**, and **Supplementary Table S5**. The top three categories in positive ion mode were amino acids and their derivatives (22.5%), benzene and its substituted derivatives (14.02%), and heterocyclic compounds (10.56%). The top three categories in negative ion mode were organic acids and their derivatives (16.75%), benzene and its substituted derivatives (14.2%), and amino acids and their derivatives (13.69%). The additional categories are presented in S2 **Supplementary Figure S4**. For ease of reference, all metabolite names used in subsequent analyses were assigned continuous IDs prefixed with “M_.”

### Candidate biomarkers for molecular classification

**Figure 1:**
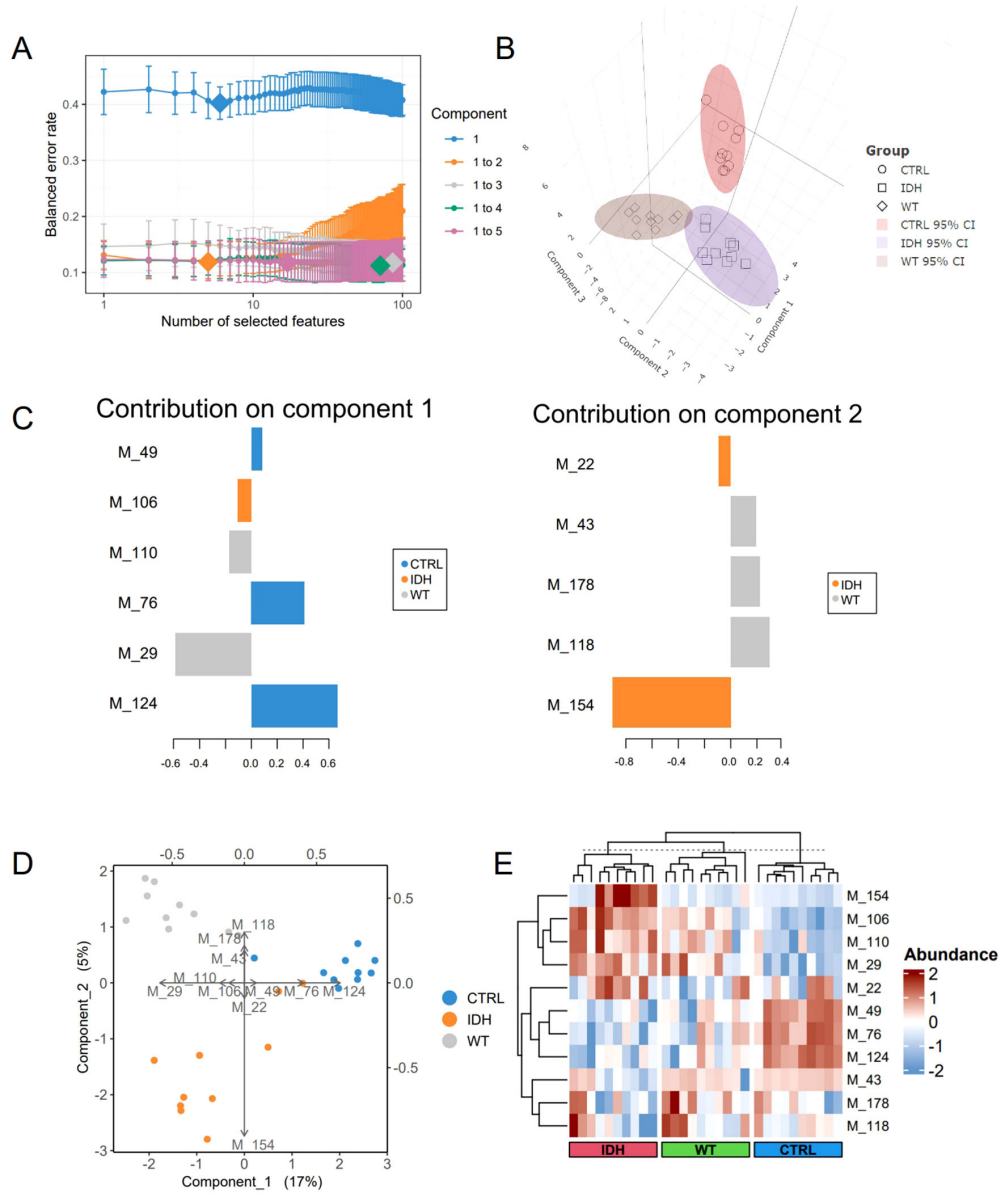
Results of model tuning and assessment. **(A)**, Tuning results for the sPLS-DA model. Each colored line represents the balanced error rate (y-axis) for each component across all tested variables (x-axis), with the standard deviation indicated. **(B)**, Sample visualizations based on three components. The samples are projected into the space spanned by the first three components, with 95% ellipse confidence intervals around each sample class. **(C)**, Contributions of characteristic metabolites. Metabolites are ranked according to their loading weights (most important at the bottom to least important at the top), represented as a bar plot. The color indicates the group to which the biomarker primarily contributes. **(D)**, Biplot from the sPLS-DA model after variable selection. The plot highlights which metabolite is related to a specific group. **(E)**, Clustered heatmap of the markers. Clustering of samples was performed via an unsupervised method (Euclidean distance).

Based on the tuning results, the optimal configuration for the final model comprised two components (**Figure 1A**), with component 1 utilizing six variables and component 2 utilizing five variables (**Figure 1C**). These biomarkers were as follows: Glycerophosphocholine (M_22), 5-aminolevulinic acid (M_124), asparagine (M_43), dulcitol (M_29), gamma-glutamylcysteine (M_118), ictaconic acid (M_154), L-aspartic acid (M_49), L-tryptophan (M_110), L-valine (M_106), lysophosphatidylcholine (18:0/0:0) (M_178), and sarcosine (M_76). Visualization of the samples demonstrated that the final model exhibited robust discriminative power among nontumor, IDH-mutant, and IDH-wild-type patients (**Figure 1B**). Specifically, the metabolites in component 1 were primarily distinguished between the WT and CTRL groups, whereas those in component 2 were mainly responsible for subclassifying the IDH and WT groups (**Figures 1C, D**). Clear separation of all three groups was observed in the unsupervised-clustered heatmap of the metabolites, characterized by distinct metabolic signatures (**Figure 1E**). Detailed information on these metabolites, including their abundances, is provided in S1 **Supplementary Table S2**, and the significance of the differences between the groups is illustrated in S2 **Supplementary Figure S5**.

**Figure 2:**
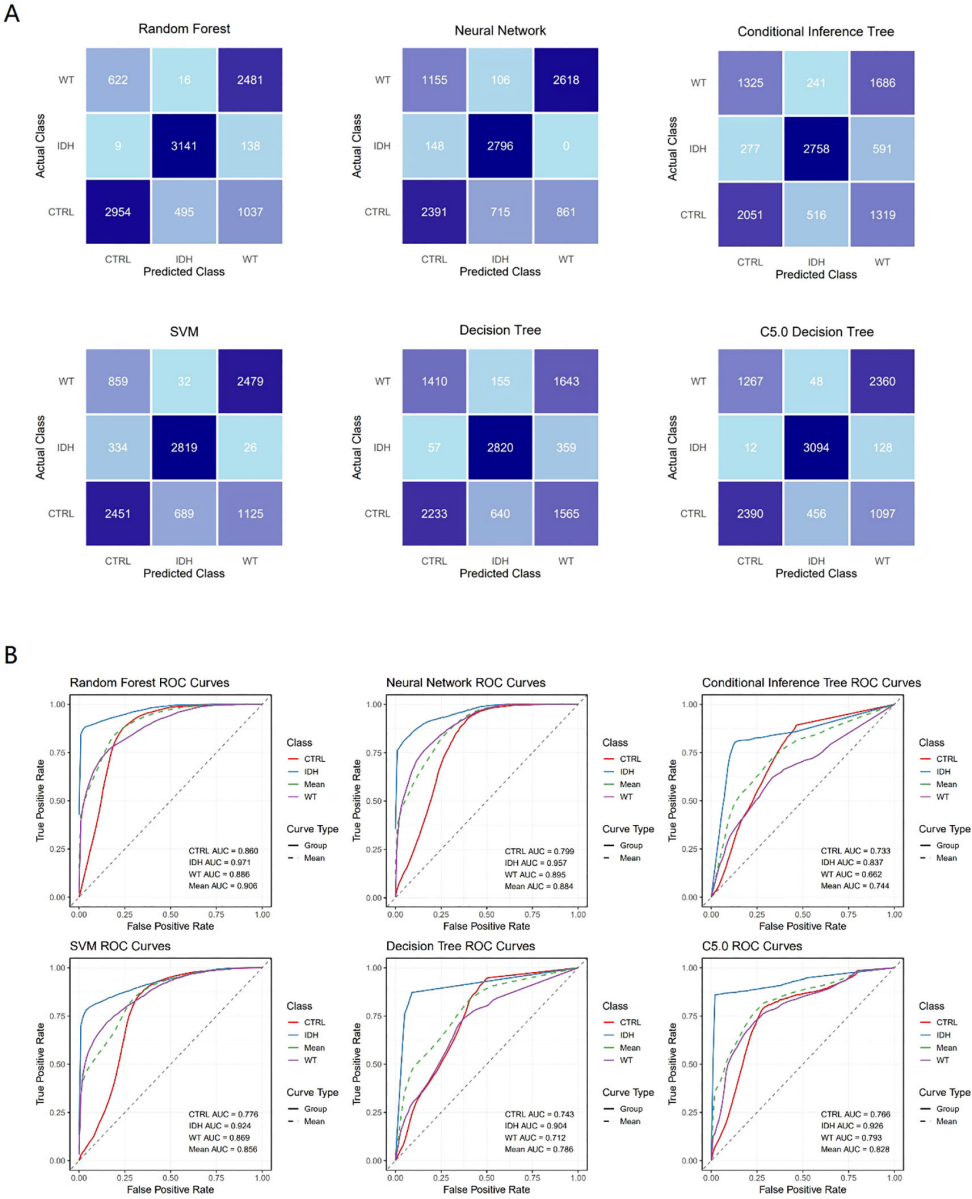
Validation results of classical models. The confusion matrices **(A)** and the ROC curves **(B)** of six classical machine learning models (Decision Tree, Random Forest, Neural Network, Conditional Inference Tree, C5.0 Decision Tree, and Support Vector Machine) applied to classification models built using the top three ranked (by frequency) biomarkers. The validation process employed bootstrap resampling to ensure assessment stability.

The performance of six validation models was established using top 3 biomarkers, itaconic acid, dulcitol, and 5-aminolevulinic acid, and was evaluated using confusion matrices (**Figure 2**). The Random Forest model demonstrated the highest accuracy of 0.787 (95% CI: 0.780–0.795), followed by the Neural Network (0.723, 95% CI: 0.715–0.732), C5.0 (0.723, 95% CI: 0.714– 0.731), SVM (0.717, 95% CI: 0.708–0.725), Decision Tree (0.615, 95% CI: 0.606–0.625), and Conditional Inference Tree (0.603, 95% CI: 0.594–0.613). The Random Forest model also exhibited the highest Kappa value (0.681), indicating better agreement beyond chance, while the Conditional Inference Tree had the lowest (0.405). Sensitivity, specificity, and F1 scores varied across models and classes, with the Random Forest generally showing superior performance in these metrics as well. There was significant non-randomness in the prediction errors of all models (*p* value of McNemar’s Test < 2.2e-16) (**Table 1**).

**Table 1:** The results of the six validation models established using top three ranked (by frequency) biomarkers.

Model	Random Forest			Neural Network			Conditional Inference Tree			SVM			Decision Tree			C5.0 Decision Tree		
Group	**CTRL**	**IDH**	**WT**	**CTRL**	**IDH**	**WT**	**CTRL**	**IDH**	**WT**	**CTRL**	**IDH**	**WT**	**CTRL**	**IDH**	**WT**	**CTRL**	**IDH**	**WT**
Sensitivity	0.824	0.860	0.679	0.647	0.773	0.753	0.562	0.785	0.469	0.673	0.796	0.683	0.604	0.780	0.461	0.651	0.860	0.658
Specificity	0.790	0.980	0.912	0.778	0.979	0.828	0.742	0.880	0.782	0.747	0.951	0.876	0.693	0.943	0.786	0.784	0.981	0.819
PPV	0.659	0.955	0.795	0.603	0.950	0.675	0.528	0.761	0.519	0.575	0.887	0.736	0.503	0.871	0.512	0.606	0.957	0.642
NPV	0.902	0.933	0.849	0.809	0.895	0.875	0.767	0.894	0.746	0.818	0.906	0.845	0.772	0.896	0.749	0.815	0.934	0.829
Precision	0.659	0.955	0.795	0.603	0.950	0.675	0.528	0.761	0.519	0.575	0.887	0.736	0.503	0.871	0.512	0.606	0.957	0.642
Recall	0.824	0.860	0.679	0.647	0.773	0.753	0.562	0.785	0.469	0.673	0.796	0.683	0.604	0.780	0.461	0.651	0.860	0.658
F1 score	0.732	0.905	0.732	0.624	0.852	0.712	0.544	0.772	0.492	0.620	0.839	0.708	0.549	0.823	0.485	0.628	0.906	0.650
DR	0.271	0.288	0.228	0.222	0.259	0.243	0.191	0.256	0.157	0.227	0.261	0.229	0.205	0.259	0.151	0.220	0.285	0.218
DP	0.412	0.302	0.286	0.368	0.273	0.360	0.361	0.337	0.302	0.394	0.294	0.312	0.408	0.297	0.295	0.363	0.298	0.339
Balanced Accuracy	0.807	0.920	0.795	0.713	0.876	0.790	0.652	0.832	0.625	0.710	0.873	0.779	0.648	0.861	0.623	0.718	0.920	0.739
Accuracy (95% CI)	0.787 (0.780–0.795)			0.723 (0.715–0.732)			0.603 (0.594–0.613)			0.717 (0.708–0.725)			0.615 (0.606–0.625)			0.723 (0.714–0.731)		
P	< 2.2e-16			< 2.2e-16			< 2.2e-16			< 2.2e-16			< 2.2e-16			< 2.2e-16		
Kappa	0.681			0.585			0.405			0.575			0.422			0.584		

P, p value of McNemar’s Test; SVM, support vector machine; DR, detection rate; DP, detection prevalence; PPV, positive predictive value; NPV, negative predictive value.

### Candidate biomarkers for survival risk prediction

**Figure 3:**
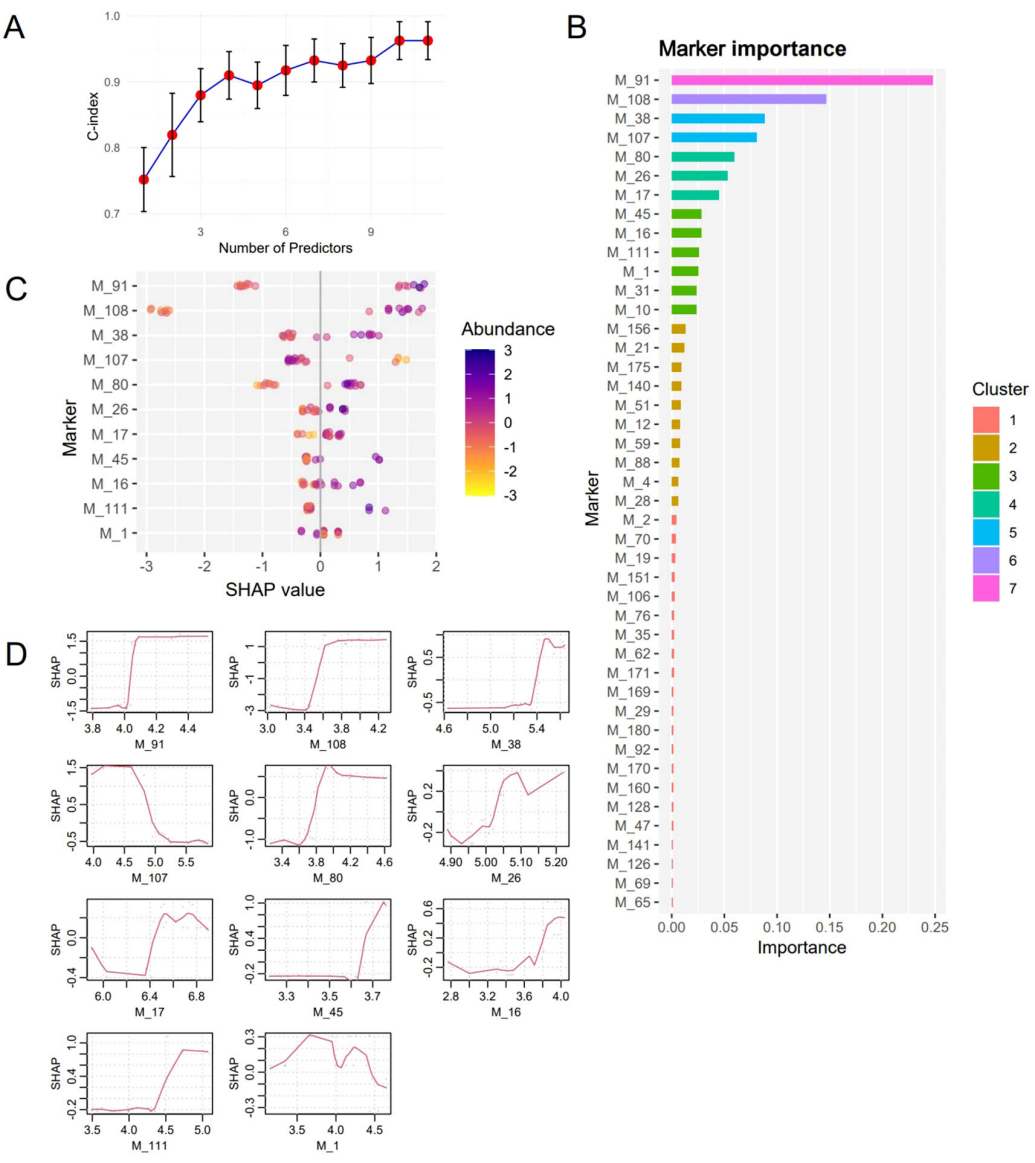
Evaluation of biomarkers for predicting survival risk. **(A)**, C-index of the survival risk prediction models using different combinations of markers. The biomarkers were added to the model for training in descending order of contribution values. **(B)**, Importance of biomarkers for molecular subtypes calculated via the XGBoost algorithm and Cox proportional hazards regression model. **(C)**, SHapley Additive exPlanations (SHAP) contributions of different features. Each point represents one case and is colored based on its abundance. **(D)**, These plots represent how the SHAP feature contributions depend on the biomarker values.

**Figure 4:**
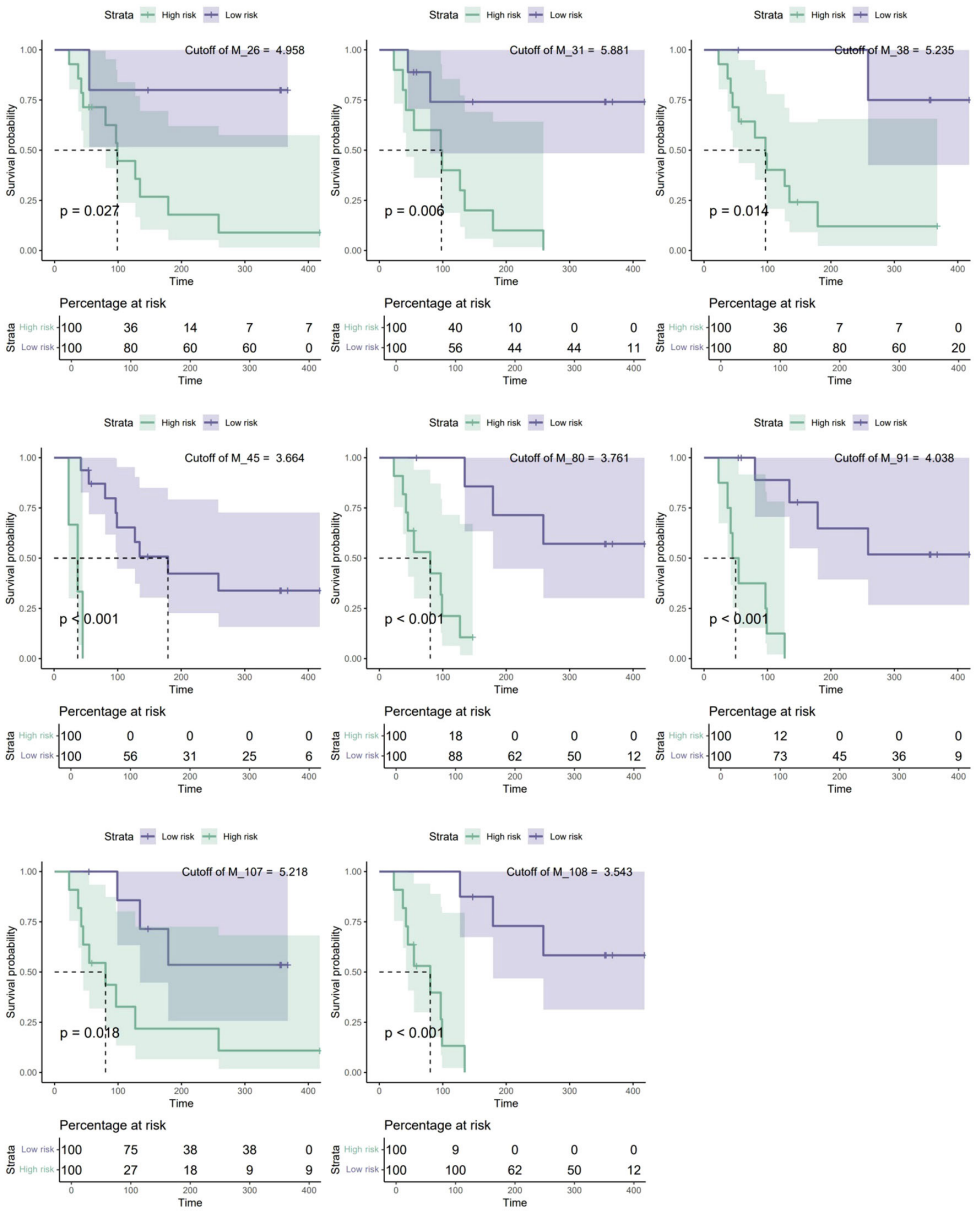
Eight biomarkers significantly associated with survival risk. Among the 11 biomarkers used to establish the survival prediction model, 8 biomarkers showed significant correlations with survival risk. Specifically, M_38, M_45, M_91, M_107, and M_111 also had a significant impact on overall survival in the validation dataset. * A high abundance of M_107 indicates low risk, whereas a low abundance indicates high risk.

**Figure 5:**
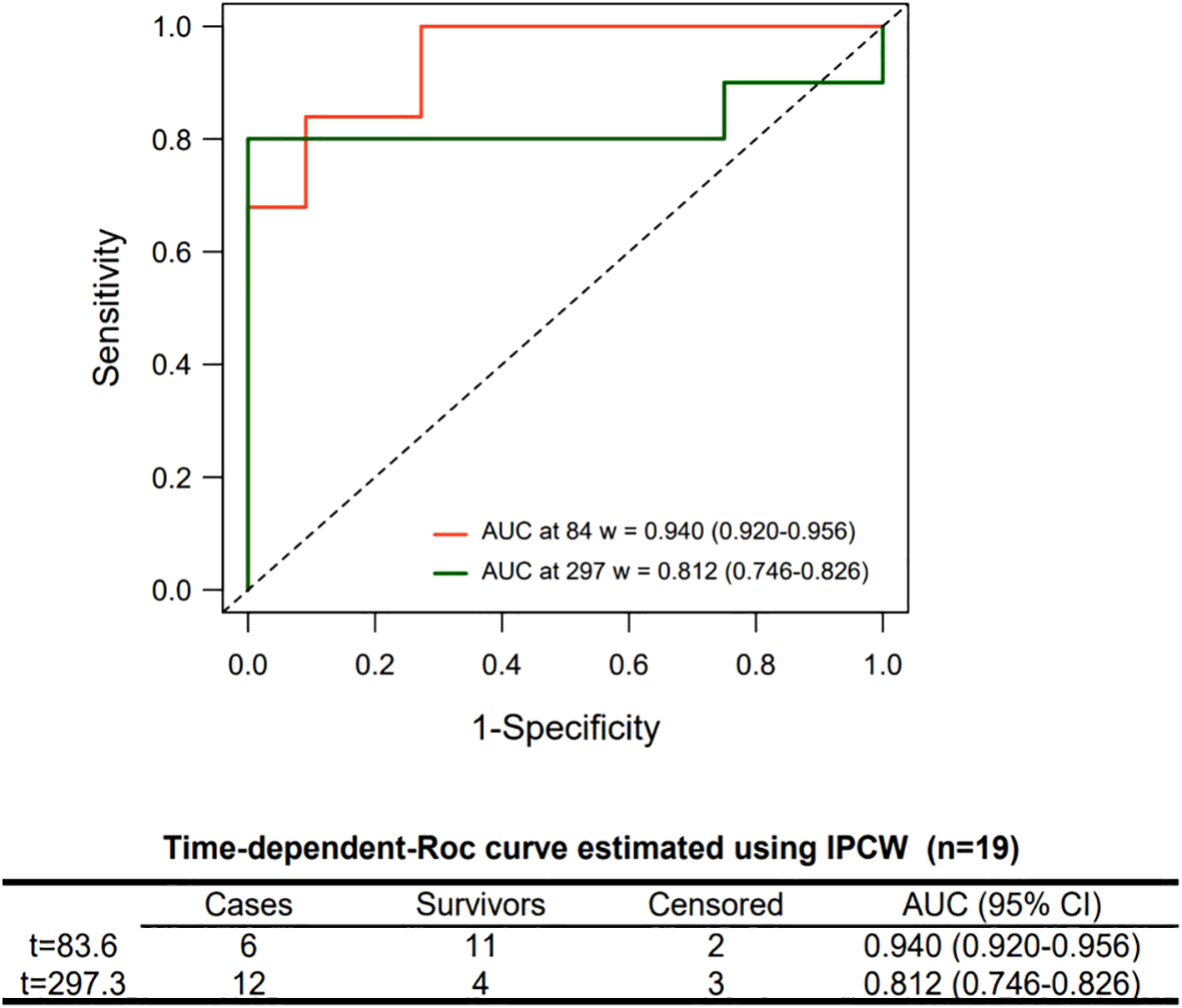
Time-dependent ROC curve of the Cox proportional hazards regression model. The Cox model was established using top 2 biomarkers. Average area under the curves (AUCs) and 95% confidence intervals (CIs) were obtained via bootstrap resampling.

Patient No. 1 in the IDH group was lost to follow-up and was thus excluded from this analysis. Nineteen samples (9 from the IDH group and 10 from the WT group) were used for this analysis. After 88 epochs, the XGBoost model stopped converging, and 44 biomarkers were identified. Due to the sample size constraints, the model could not converge effectively when incorporating more than 11 variables. In the time-dependent ROC curve analysis, the metabolites were restricted to 7 because 5-fold cross-validation was employed. Iterative modeling revealed that the top 11 biomarkers had a C-index of 0.962 (95% CI: 0.934-0.991) (**Figure 3A**). The 11 biomarkers were cholic acid (M_91), citrulline (M_108), L-tyrosine (M_38), nicotinamide-adenine dinucleotide (M_107), uric acid (M_80), xylose (M_26), creatine (M_17), L-histidine (M_45), hydrocortisone (M_16), uridine-5’-diphosphoglucuronic acid (M_111), and butanoic acid (M_1). The differences in these metabolites among each group are presented in S2 **Supplementary Figure S6**. Additionally, SHapley Additive exPlanations (SHAP) values and feature importance revealed that the importance of the biomarkers gradually decreased as the ranking increased (**Figures 3B–D**). The significance tests of survival curves revealed that all 11 biomarkers, except for M_1, M_16, and M_17, had significant impact on the survival of patients with GBM (**Figure 4**). Seven of these markers were successfully validated in an independent dataset for their association with survival risk, and five of these (M_38, M_45, M_91, M_107, and M_111) validated markers were consistent with the findings of our study (**Supplementary Figure S7**). However, the validation dataset lacks samples with IDH mutations, which could be an important reason for the discrepancies. The final Cox proportional hazards regression model was established using the top 2 biomarkers, cholic acid and citrulline, through fivefold cross-validation. The time-dependent ROC curve demonstrated that the Cox model achieved area under the curve (AUC) values of 0.942 and 0.826 at 84 and 297 weeks, respectively (**Figure 5**).

## Discussion

Similar studies have identified serum biomarkers for GBM diagnosis and survival risk prediction, including arginylproline, 5-hydroxymethyluracil, arginine, methionine, and kynurenate (13, 14). However, these metabolites were not detected in this study. This discrepancy may be attributed to the heterogeneity between plasma and tissue and variations in detection methods. Our metabolomic results were derived directly from GBM tissues and subjected to strict screening criteria, which increased the tumor specificity of the identified biomarkers. However, further validation is needed if these biomarkers are to be used in combination for fluid-based diagnosis. Although several biomarkers exhibited different mean abundances across the three groups (nontumor patients, IDH-mutant GBM patients, and IDH-wildtype GBM patients), their distributions significantly overlapped. This finding indicates that single metabolites cannot meet the requirements for trichotomous classification, and a classification model based on multiple biomarkers needs to be established to obtain a more stable prediction performance; this is also the reason why we used multiple classic algorithms to validate the representativeness of the biomarkers.

In this study, 11 metabolites were identified as suitable for combined use in building a classification model. Several of these metabolites were previously identified in GBM tissue metabolomics studies, with their reported levels aligning consistently with our findings, such as glycerophosphocholine (M_22) (16), L-tryptophan (M_110) (17), asparagine (M_43) (18), and LysoPC(18:0) (M_178) (19). Although direct clinical evidence remains limited for the abundance of other markers in glioma tissues, existing studies demonstrate their significant associations with gliomas or other tumor types. Asparagine (M_43) is synthesized from aspartate and glutamine by asparagine synthetase. Its levels in the IDH group were significantly lower than those in the WT group in this study, which may partially explain why glioma patients with low asparagine levels have better survival rates (20). Dulcitol (M_29) participates in galactose metabolism. Currently, there is limited clinical research on its abundance in human tumor tissues. *In vitro* and animal experiments have shown that it has anti-tumor effects (21, 22). Our results indicate that its levels are significantly elevated in GBM tissues, including both the WT and IDH groups. The mechanism of dulcitol elevation and its role in GBM require further investigation. Bao et al.’s study demonstrated a significant positive correlation between valine (M_106) in cerebrospinal fluid and the incidence of glioblastoma (23). In this study, valine levels were elevated in both the IDH and WT groups, further strengthening its correlation with GBM. Sarcosine (M_76) levels are increased in many types of tumors and associated with invasiveness (24, 25). Nervertheless, there are currently no clinical studies clearly investigating changes in sarcosine levels in GBM tissues. *In vitro* experiments have shown that glioma cells convert glycine to sarcosine through glycine-N-methyltransferase, leading to elevated sarcosine levels in the microenvironment (26, 27). Sarcosine competitively inhibits the GlyT1 transporter of dendritic cells (DCs), thereby upregulating the expression of COX-1 and CXCR2, and promoting DCs’ migration to the tumor area (26). Interestingly, our results showed that sarcosine in GBM tissues was significantly lower than that in normal brain tissues. Further research is needed to determine whether this metabolic change is associated with DC-mediated immune responses. Glioma cells do not directly synthesize itaconic acid (28). Instead, this metabolite predominantly originates from metabolic byproducts of M1-polarized macrophages (28, 29). Wang et al.’s study demonstrated that IDH-mutant glioma tissues contain a significantly higher proportion of M1-polarized macrophages compared to that in wild-type gliomas (30). This result may explain the reason for the increased levels of itaconic acid in the IDH group in our study.

Biomarkers for survival prediction have been demonstrated to be closely linked to GBM progression. For example, creatine has been used extensively to facilitate the migration and proliferation of GBM cells, although its impact on survival risk was not significant in this study (31). Elevated cholic acid levels increase the invasive capacity and drug resistance of GBM cells (32). The uric acid concentration is correlated with the extent of necrosis in the GBM mass. High glutathione levels are intimately associated with glioblastoma cell survival (33). Furthermore, nicotinamide adenine dinucleotide (NAD+) augments the tumor-suppressing capabilities of immune cells and increases chemosensitivity (34, 35). Consistently, our results revealed that GBM patients with low NAD+ levels presented a significantly increased survival risk. Interestingly, L-histidine has been reported to have anticancer properties (36), while our results suggest that high levels of L-histidine may increase the survival risk for GBM patients. This inconsistent finding requires further confirmation. The remaining biomarkers, such as citrulline and uridine-5’-diphosphoglucuronic acid, have not yet been reliably associated with GBM. Nevertheless, previous studies have shown that citrullinated histone H3 (CitH3) plays a pivotal role in the formation of neutrophil extracellular traps (NETs), which can accelerate glioblastoma progression (37). The accumulation of uridine-5’-diphosphoglucuronic acid has also been implicated in cancer metastasis (38). The above studies support the reliability of biomarkers, and our study is the first to reveal the associations between these biomarkers and survival risk.

There are several limitations that need to be addressed. Despite evaluating the validation models via bootstrap-resampling, the small sample size may still lead to overfitting. To mitigate overfitting, we restricted the number of variables in both classification and survival models to 1/10 of the sample size (e.g., 3 biomarkers for classification models and 2 for survival analysis) and focused only on top-ranked biomarkers. However, even with these precautions, the reported AUC values should be referenced with caution as they may still be overestimated due to the inherent limitations associated with small sample sizes. While partial validation was performed using an independent dataset, the heterogeneity of the external cohort (e.g., lack of IDH-mutant cases) may limit the generalizability of our findings. Future studies should validate these biomarkers through large-scale targeted metabolomics. The biomarkers identified here are tissue-derived, and their expression levels and diagnostic thresholds in biofluids (e.g., cerebrospinal fluid) require further investigation. Additionally, the biological roles of certain markers (e.g., sarcosine) in tumor microenvironments remain unclear and necessitate functional studies to elucidate their mechanisms. Untargeted metabolomics has limited coverage which potentially omits previously reported metabolites by other studies. Integrating targeted assays and multi-omics approaches in our future studies will strengthen the biomarker framework.

In summary, this exploratory work highlights potential metabolic biomarkers for GBM subtyping and survival risk. However, clinical application requires multicenter validation with larger cohorts and mechanistic studies to clarify the biological basis of metabolic reprogramming.

## Data Availability

The original contributions presented in the study are included in the article/**Supplementary Material**. Further inquiries can be directed to the corresponding author.
