# Supplementary material for: Nontargeted metabolomics uncovering metabolite signatures in glioblastoma: a preliminary study on candidate biomarker discovery for IDH subtyping and survival prediction
Source: Front Oncol. 2025 May 8;15:1568040. doi: 10.3389/fonc.2025.1568040 (PMC12095168; doi:10.3389/fonc.2025.1568040)
Supplement: Supplementary file 2 [file Table2.docx]

**1 Reagents and instruments**

| **Name** | **CAS Number** | **Purity** | **Brand** | **Product Code** |
| --- | --- | --- | --- | --- |
| Methanol | 67-56-1 | HPLC Grade | Merck | 1.06007.4008 |
| Acetonitrile | 1975/5/8 | HPLC Grade | Merck | 1.00030.4008 |
| Acetic Acid | 64-19-7 | HPLC Grade | Luoen | R009611-500ml |
| Ammonium Formate | 540-69-2 | HPLC Grade | Aladdin | 516961-100G |
| Ammonia Water | 1336-21-6 | HPLC Grade | Aladdin | 221228-100ML-A |
| Formic Acid | 64-18-6 | HPLC Grade | Aladdin | 695076-100ML |
| Standards | - | >98% | isoreag/TRC/  TCI/Sigma | - |

**Table 1:** List of reagents.

| **Name** | **Model** | **Brand** | **Origin** |
| --- | --- | --- | --- |
| Mass Spectrometer | TripleTOF 6600+ | SCIEX | Foster City, CA, USA |
| Ultra-High-Performance Liquid Chromatograph | LC-30A | Shimadzu | Japan |
| Centrifuge | 5424R | Eppendorf | Hamburg, Germany |
| Thermostatic Metal Mixer | MU-G02-0448 | Hangzhou Miou Instrument Co., Ltd. | Hangzhou, China |
| Analytical Balance (0.00001 g) | MS105DΜ | Mettler Toledo | Zurich, Switzerland |
| Centriconcentrator | CentriVap | LABCONCO | Missouri Kansas, USA |
| Vortex Mixer | VORTEX-5 | Kyllin-Bell | Haimen, China |
| Ultrasonic Cleaner | KQ5200E | Kunshan Ultrasonic Instrument Co., Ltd. | Kunshan, China |
| Pipette | Research plus | Eppendorf | Hamburg, Germany |
| Automated Workstation | Biomek i5 | Beckman Coulter | California, USA |
| Sealing Machine | Mini HES | Monad | Suzhou, China |

**Table 2:** Equipment List.

**2 Chromatography-Mass Spectrometry Acquisition Conditions**

(1) Chromatographic Column: Waters ACQUITY Premier HSS T3 Column, 1.8 µm, 2.1 mm × 100 mm

(2) Mobile phase A: 0.1% formic acid in water; mobile phase B: 0.1% formic acid in acetonitrile

(3) Column temperature: 40 °C; flow rate: 0.4 mL/min; injection volume: 4 μL

| **Time (min)** | **A (%)** | **B (%)** |
| --- | --- | --- |
| 0 | 95 | 5 |
| 2 | 80 | 20 |
| 5 | 40 | 60 |
| 6 | 1 | 99 |
| 7.5 | 1 | 99 |
| 7.6 | 95 | 5 |
| 10 | 95 | 5 |

**Table 3:** T3 chromatographic column mobile phase gradient conditions.

| **English Name** | **Chinese Name** | **ESI+** | **ESI-** |
| --- | --- | --- | --- |
| Duration (min) | Acquisition Time (min) | 10 | 10 |
| IonSpray Voltage (V) | Ionization Voltage (V) | 5000 | -4000 |
| Temperature (°C) | Ion Source Temperature (°C) | 550 | 450 |
| Ion Source Gas1 (psi) | Nebulizing Gas (psi) | 50 | 50 |
| Ion Source Gas2 (psi) | Heater Gas (psi) | 60 | 60 |
| Curtain Gas (psi) | Curtain Gas (psi) | 35 | 35 |
| Declustering Potential (V) | Declustering Potential (V) | 60 | -60 |
| MS1 Collision Energy (V) | MS1 Collision Energy (V) | 10 | -10 |
| MS2 Collision Energy (V) | MS2 Collision Energy (V) | 30 | -30 |
| Collision Energy Spread (V) | Collision Energy Spread (V) | 15 | 15 |
| MS1 TOF Masses (Da) | MS1 Mass Range (Da) | 50~1000 | 50~1000 |
| MS2 TOF Masses (Da) | MS2 Mass Range (Da) | 25~1000 | 25~1000 |
| MS1 Accumulation time (s) | MS1 Accumulation Time (s) | 0.2 s | 0.2 s |
| MS2 Accumulation time (s) | MS2 Accumulation Time (s) | 0.04 s | 0.04 s |
| Candidate Ions | Candidate Ions | 18 | 18 |
| Exclude Former Target Ions | Ion Exclusion Logic | Always, For 3 Seconds, After 3 Occurrences | Always, For 3 Seconds, After 3 Occurrences |

**Table 4:** Mass spectrometry conditions for the AB TripleTOF 6600 instrument.

**3 Sample Quality Control Analysis**

During instrument analysis, a QC sample is typically inserted after every 10 analytical samples to monitor the reproducibility of the analytical process.

**3.1 Total Ion Chromatogram (TIC)**

By overlaying and analyzing the total ion chromatograms (TICs) of different QC samples from mass spectrometry analysis, the repeatability of metabolite extraction and detection, i.e., technical repeatability, can be assessed. High instrument stability provides an important guarantee for data repeatability and reliability.


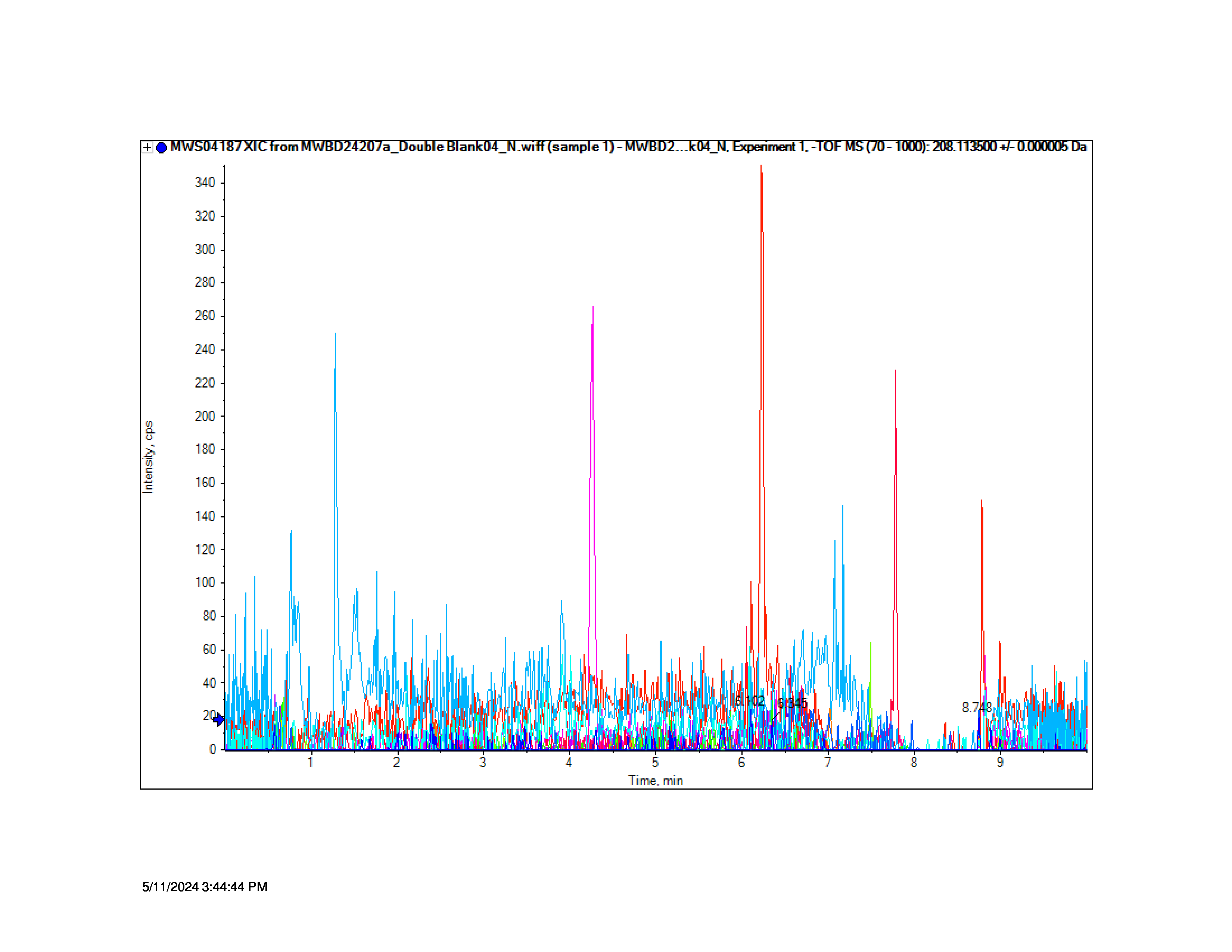

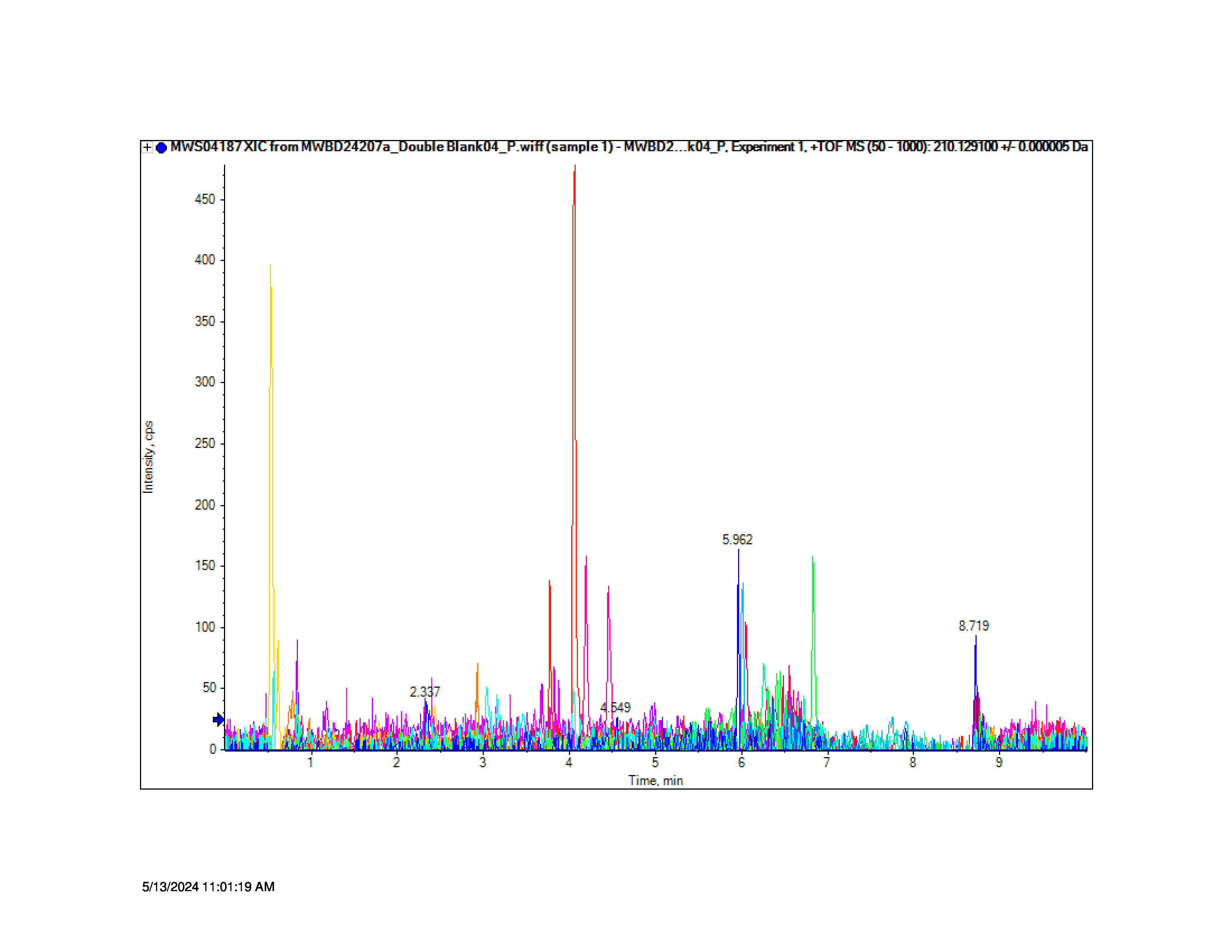


**Fig. 1:** Overlay plot of TICs from QC sample mass spectrometry analysis. The results show high overlap of the total ion current curves, indicating consistency in retention time and peak intensity, which suggests good signal stability for the same sample detected at different times. On the left is the positive mode, and on the right is the negative mode.

**3.2 Internal Standard Peak Detection in Blank Samples**


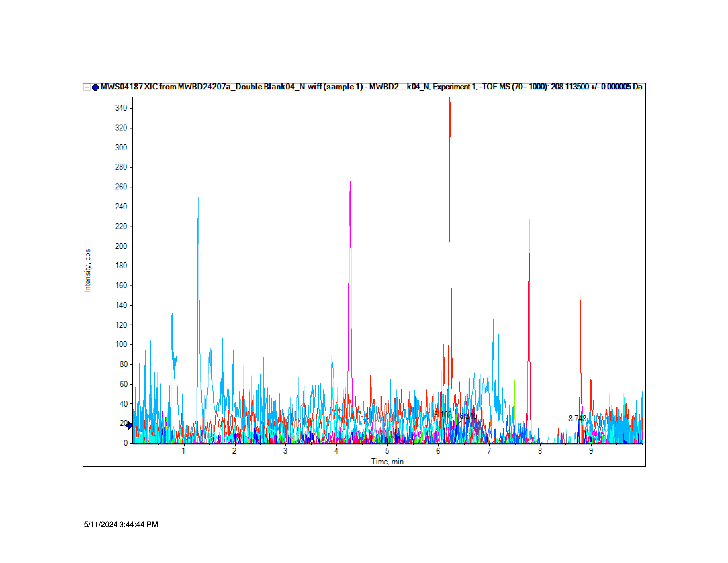

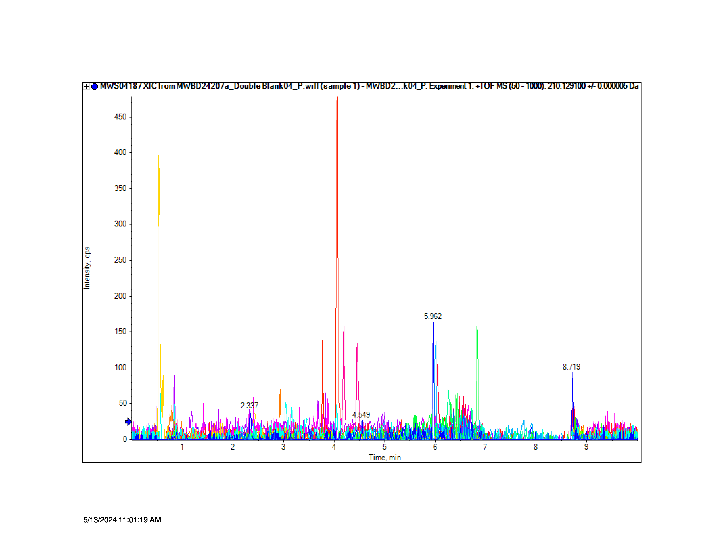


**Fig. 2:** Extracted ion chromatography (EIC) plot of internal standards in blank samples. The figures show that no significant peaks of internal standards are detected in the blank samples, indicating minimal substance residue and cross-contamination among samples within a controllable range. On the left is the positive mode, and on the right is the negative mode.

**3.3 QC Sample Correlation Analysis**

Pearson correlation analysis was performed on the QC samples. A higher QC sample correlation (| r | closer to 1) indicates better stability throughout the detection process and higher data quality.


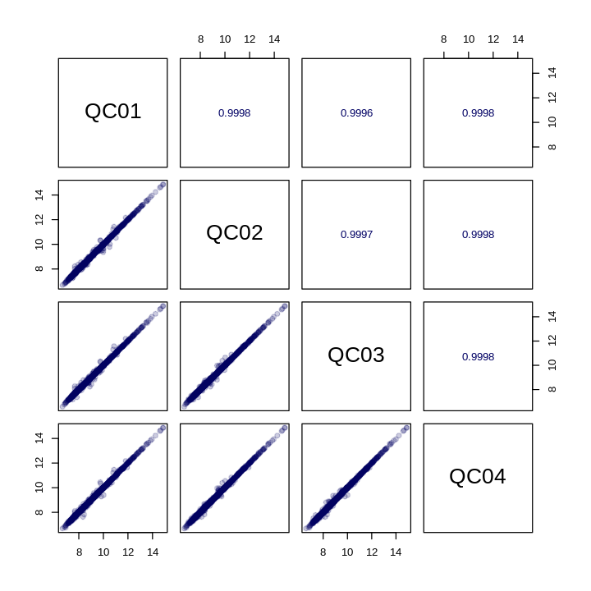

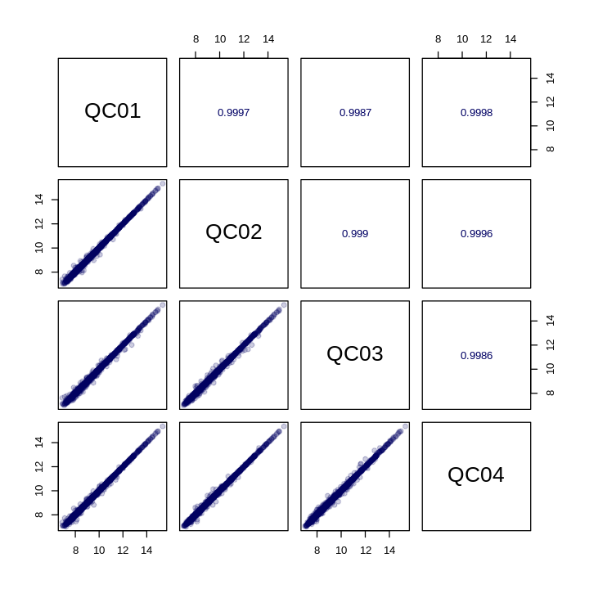


**Fig. 3:** QC sample correlation plot. Pearson correlation analysis was performed on the QC samples. A higher QC sample correlation (| r | closer to 1) indicates better stability throughout the detection process and higher data quality. The diagonal squares represent the QC sample names; the squares in the lower left corner of the diagonal represent the scatter plots of the corresponding QC sample correlations, with the metabolite content (log-transformed) on both the x- and y-axes; each point in the plot represents a metabolite; and the squares in the upper right corner of the diagonal represent the Pearson correlation coefficients for the corresponding QC samples.

**3.4 Internal Standard Stability in QC Samples**

| **Index** | **Q1 (Da)** | **RT (min)** | **CV** |
| --- | --- | --- | --- |
| MWS1055 | 126.061 | 4.4 | 0.0086 |
| MWS04428 | 144.1114 | 6.31 | 0.0089 |
| MWS3085 | 278.9903 | 4.25 | 0.0171 |
| MWS04187 | 208.1143 | 2.36 | 0.0186 |

**Table 5:** Internal standard stability of the QC samples. Known concentrations of internal standards were added to the QC samples. Smaller response variations of internal standards indicate a more stable detection process and higher data quality.

**4 Metabolite Identification**


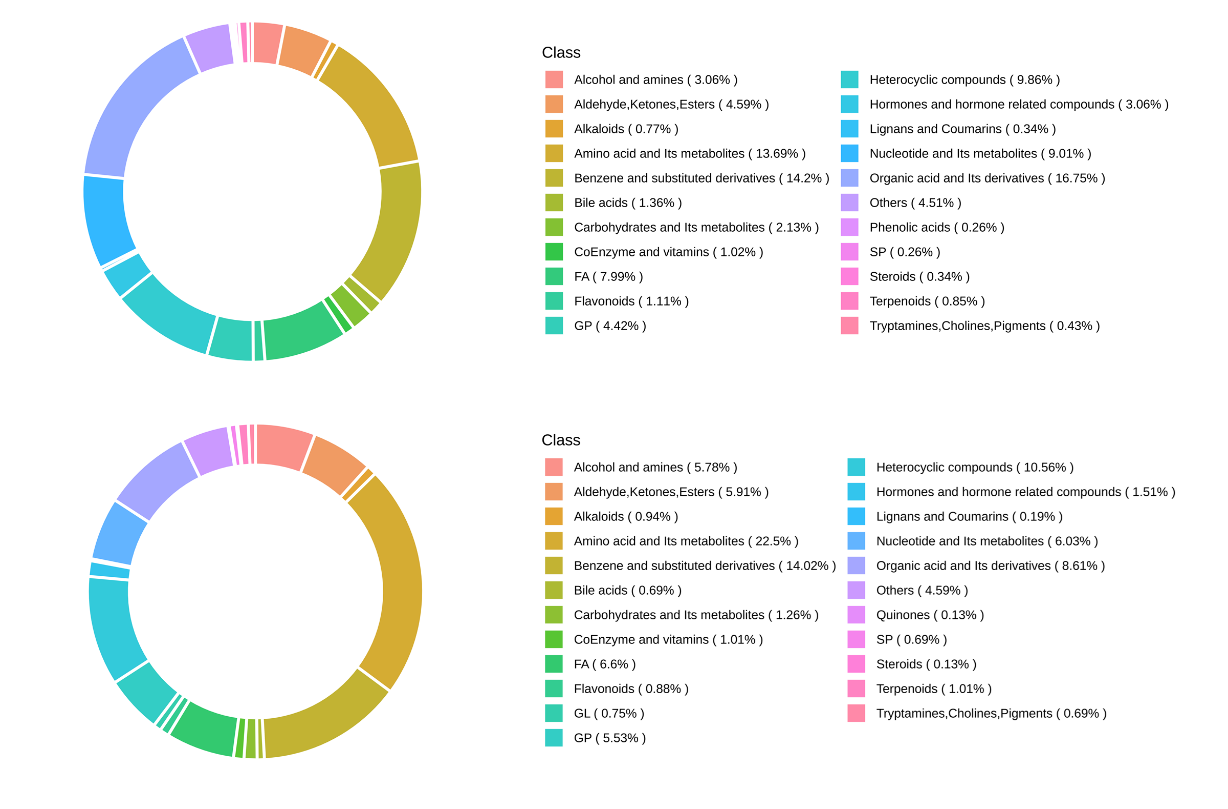


**Fig. 4:** Circular Chart of the Metabolite Category Composition. In this study, a total of 2,533 metabolites were detected from 30 selected samples.


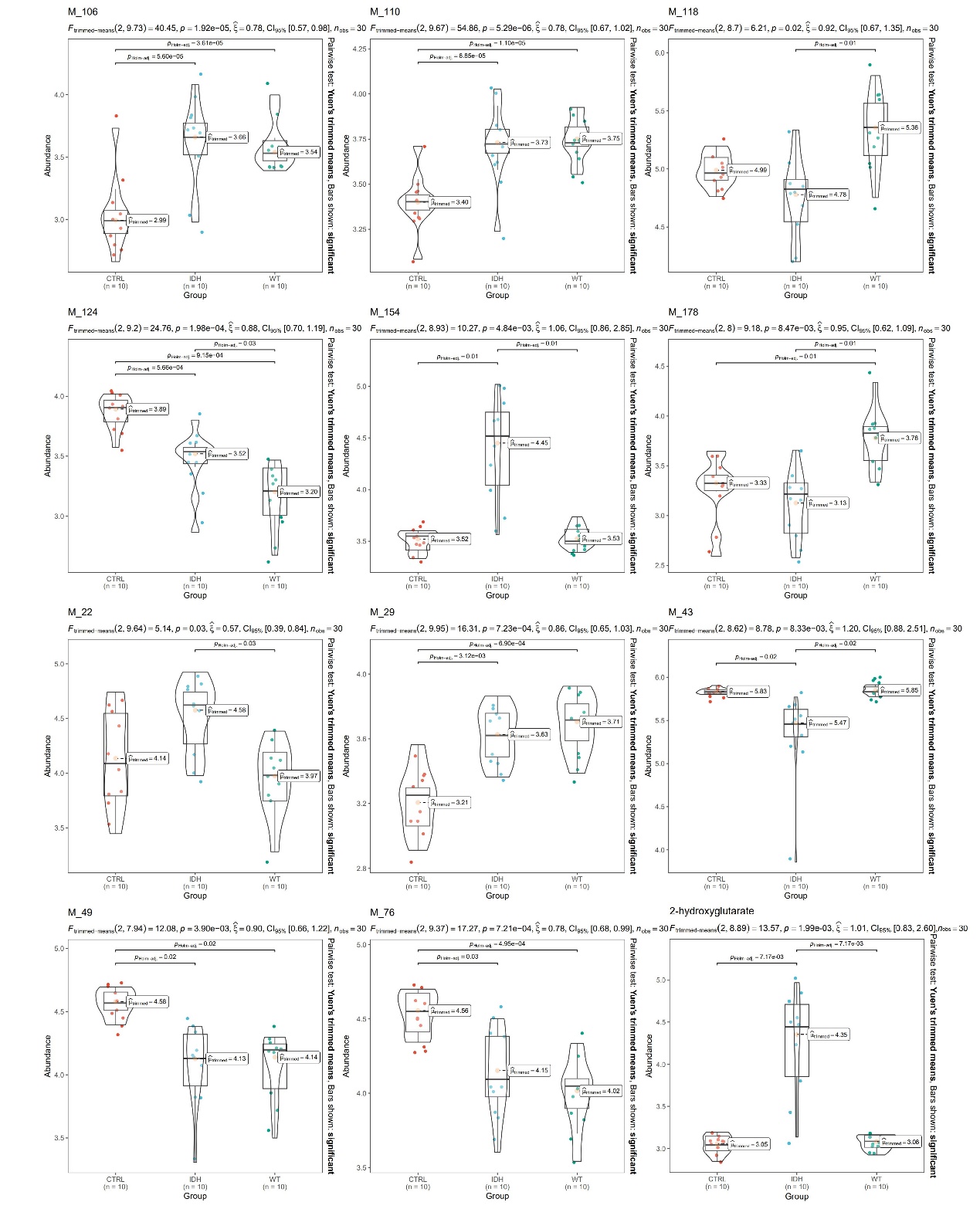


**Fig. 5:** Comparisons of candidate markers used for molecular subtype prediction between each group. The upper and lower edges of the box represent the upper and lower quartiles, respectively, whereas the middle line represents the median. The violin plot is drawn based on the data distribution. The mean is indicated by a dot in yellow (this dot does not represent the metabolite abundance value). The adjusted p values for comparisons between groups are shown above (when the adjusted p value was less than 0.05). The information from the Bayes factor analysis is located at the bottom.


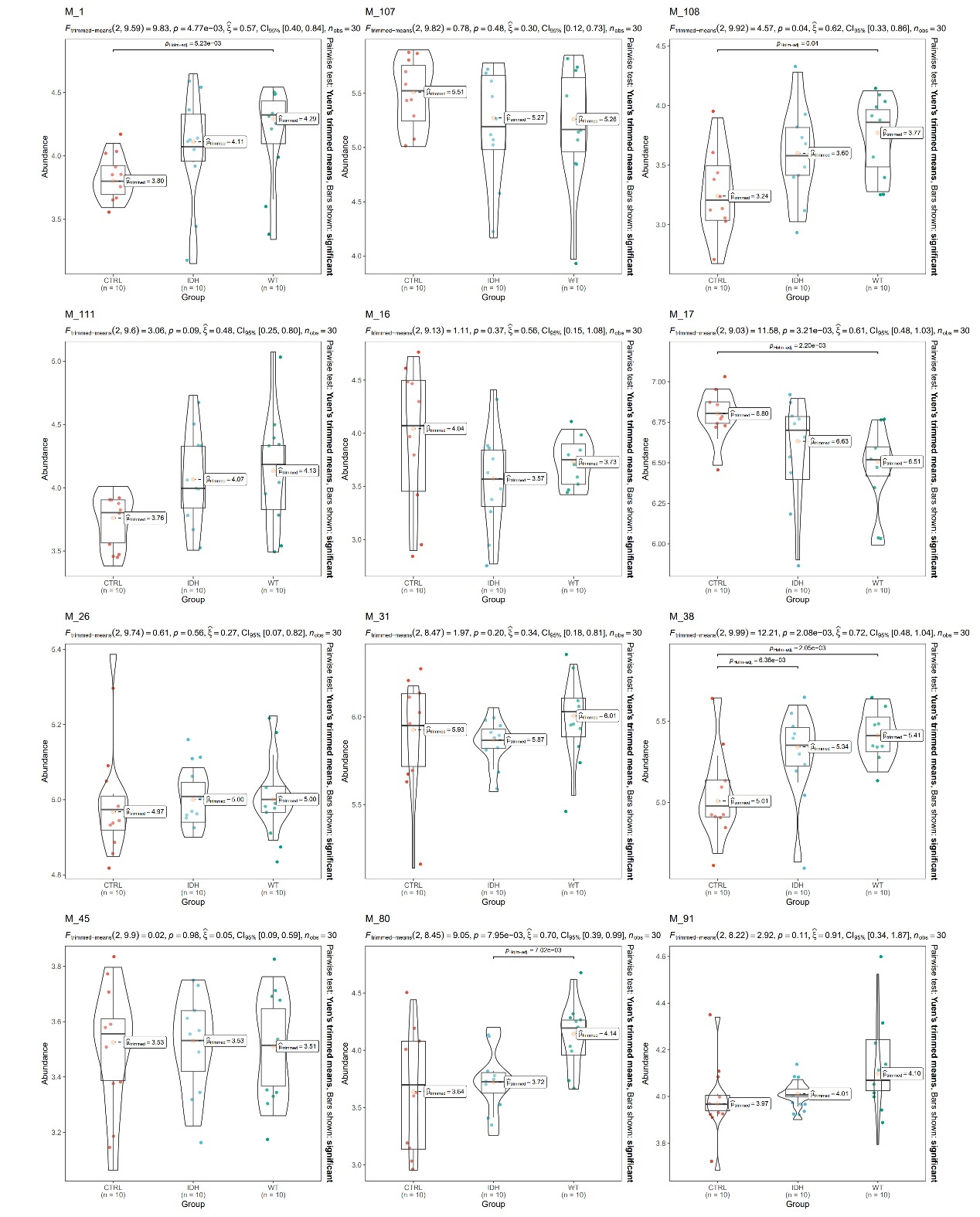


**Fig. 6:** Comparisons of candidate markers used for survival risk prediction between each group.


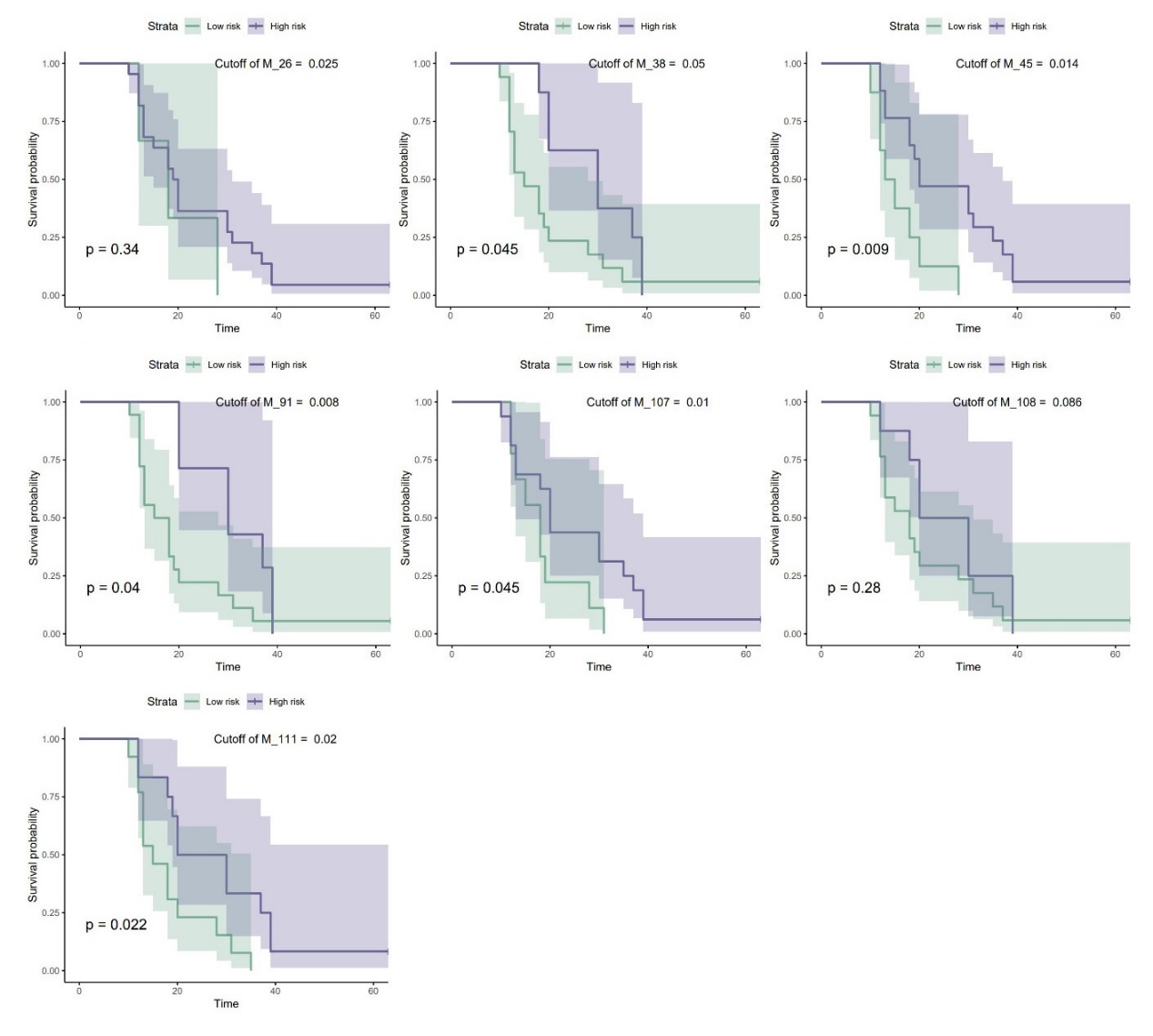

**Fig. 7:** Validation of the prognostic biomarkers in an independent dataset. The eight markers significantly associated with survival risk in Figure 4 of this study are validated in an independent metabolomics dataset of GBM (n = 25, n(IDH) = 1, n(WT) = 24). Except for M_26 and M108, the remaining markers show significant impact on with GBM survival risk.
